# Immunotherapy of hepatocellular carcinoma with small double-stranded RNA

**DOI:** 10.1186/1471-2407-14-338

**Published:** 2014-05-18

**Authors:** Tatyana O Kabilova, Larisa V Kovtonyuk, Evgeniy V Zonov, Elena I Ryabchikova, Nelly A Popova, Valeriy P Nikolin, Vasiliy I Kaledin, Marina A Zenkova, Valentin V Vlassov, Elena L Chernolovskaya

**Affiliations:** 1 Institute of Chemical Biology and Fundamental Medicine SB RAS, 8, Lavrentiev Avenue, Novosibirsk 630090, Russia; 2 Institute of Cytology and Genetics SB RAS, 10, Lavrentiev Avenue, Novosibirsk 630090, Russia

**Keywords:** Immunostimulatory RNA, Hepatocellular carcinoma, Interferon inducer

## Abstract

**Background:**

Hepatocellular carcinoma (HCC) is one of the most common malignancies worldwide with limited therapeutic options. Since HCC has been shown to be immunogenic, immunotherapy is considered a promising therapeutic approach. Small interfering RNAs (siRNAs), depending on their structure and sequence, can trigger the innate immune system, which can potentially enhance the adaptive anticancer immune response in the tumor-bearing subjects. Immunostimulatory properties of nucleic acids can be applied to develop adjuvants for HCC treatment.

**Methods:**

The transplantable HCC G-29 tumor in male CBA/LacSto (CBA) mice was used to study the effects of immunostimulatory RNA on tumor growth. Tumor size, metastases area in different organs of mice and mouse survival rate were analyzed. Furthermore the mouse serum IFN-α levels were measured using ELISA.

**Results:**

In the present study, we found that a 19-bp RNA duplex (ImmunoStimulattory RNA or isRNA) with 3-nt overhangs at the 3′-ends of specific sequence displays immunostimulatory, antitumor, and antimetastatic activities in mice bearing HCC G-29. Our results demonstrate that isRNA strongly increases the level of interferon-α (IFN-α) by up to 25-fold relative to the level in mice injected with Lipofectamine alone (Mock), and to a lesser extent increases the level of proinflammatory cytokine interleukin-6 (IL-6) (by up to 5.5-fold relative to the Mock level), in mice blood serum. We showed that isRNA reliably (P < 0.05) inhibits primary tumor growth in mice compared to the mock group. Furthermore, injections of isRNA significantly enhanced necrotic processes in the center of the primary tumor, and decreased by twofold the width of the undifferentiated peripheral zone and the number of mitotic cells in this zone. The results showed that isRNA efficiently reduces the area of metastases in the liver, kidneys, and heart of CBA/LacSto mice with HCC.

**Conclusions:**

The obtained results clearly demonstrate immunostimulatory and antimetastatic properties of the isRNAs in mice with HCC. Consequently, this short double-stranded RNA can be considered as a potential adjuvant for the therapy of HCC.

## Background

Hepatocellular carcinoma (HCC) is one of the most common cancers and the third leading cause of cancer-related deaths worldwide [1]. The standard methods of hepatocarcinoma treatment include surgical resection, chemical therapy, radiotherapy, ultrasound ablation, or liver transplantation [2-5]. However, the high rate of recurrence after these therapeutic procedures remains a big issue in patients with HCC. In view of these facts, the necessity to use adjuvant therapy becomes urgent. Tumor pathogenesis is accompanied by disorders of the immune system, leading to the escape of the tumor from the immune response. At the same time, the immune status of an organism is impaired, and immunodeficiency is developed. Immunotherapy aims to provide a more efficient targeting of tumor cells by inducing or boosting the existing tumor-specific immune response. Immunostimulatory agents are widely used as a part of the combined therapy or as a monotherapy in the treatment of different cancers [6-9]. Moreover, HCC patients with tumors containing infiltrated tumor-specific effector T cells have a reduced risk of tumor recurrence following liver transplantation [10]. The following methods of adjuvant therapy were proposed for the treatment of HCC patients: cellular immunotherapy [11], interferon (IFN) therapy [12], and therapy using endogenous IFN inducers [13]. It should be noted that in many cases, HCC develops on a background of chronic inflammatory liver disease such as hepatitis B and C or cirrhosis. Many studies have documented that IFN therapy significantly suppresses the onset of HCC from chronic hepatitis and liver cirrhosis [14-16].

Natural and synthetic immunostimulators cause a wide variety of effects at the levels of the immunocompetent cells and an entire organism including activation of different types of immune cells (macrophages, T- and B-lymphocytes, NK-cells), induction of type I interferons and/or inflammatory cytokines synthesis by immune cells, and also cause proliferation blockage, and differentiation or apoptosis of tumor cells. The innate immune system can be activated by exogenous nucleic acids (e.g., bacterial, viral, and fungal nucleic acids) via several families of pattern recognition receptors, including endosomal Toll-like receptors (TLRs) 3/7/8/9 or cytosolic receptors: retinoic acid inducible gene-I (RIG-I)-like helicases including RIG1 and melanoma differentiation-associated gene 5 (MDA5); Interferon-induced, double-stranded RNA-activated protein kinase (PKR); 2′-5′-oligoadenylate synthase 1 and 2 (OAS); and less studied NOD-like receptors (NLRP3, NOD2) [17,18]. These receptors are responsible for the activation of the complement system, coagulation, opsonization, phagocytosis, and the induction of apoptosis and cytokine production. The role of TLRs in antitumor protection of an organism is confirmed by the fact that mutations in the genes encoding TLRs increase both the incidence of infectious diseases and the frequency of cancer [19]. The antitumor activity of TLR 3/7/8/9 agonists has been demonstrated in several tumor types including melanoma, breast cancer, renal cell carcinoma, glioblastoma, cutaneous T-cell and non-Hodgkin’s lymphomas, and basal cell carcinoma [6]. Unlike other cancers, the treatment of HCC involves not only the treatment of the carcinoma itself, but also the treatment of underlying chronic hepatitis. TLR7 and −9 agonists have great therapeutic potential against HCC because they induce the synthesis of the mediators of anti-viral and anticancer immunity such as IFN-α and IFN-γ-inducible protein 10 (IP-10), and induce a sustained increase in 2′5′-oligoadenylate synthesis [20]. In a recent phase 1 trial in patients with chronic HCV infection, TLR7 agonist isatoribine (Anadys Pharmaceuticals, Inc.) and TLR9 ligand CPG 10101 (Actilon; Coley Pharmaceutical Group, Inc.) induced an effective decrease in HCV RNA [13,21-23] and the anti-viral effect was similar to that reported for pegylated interferon monotherapy [23].

Recently, we designed a series of short double-stranded RNAs that possess pronounced antiproliferative activity in cancer cells [24,25]. The study of their sequence/activity relationships showed [25] that the introduction of substitutions in the middle part of isRNA sequence does not alter the antiproliferative activity. Disruption of GUGU motif at the 5′-end of strand 1, which is similar to the reduced version of immunostimulatory sequence according to [26], caused a minor decrease of the antiproliferative activity compared with the parent isRNA. While substitutions in the 3′-end regions of isRNA substantially reduce its biological activity. Thus, introduction of only one substitution, disrupting oligoU6 motif at the 3′-end of strand 1 entirely abolishes the antiproliferative activity. The sequence of these molecules did not have substantial homology to any human or mouse mRNAs, and these compounds were shown to have the immunostimulatory effects in culture of human adherent peripheral blood mononuclear cells (PBMCs) and in C57BL mice.

In this study we investigated the antitumor and antimetastatic properties of the selected most effective immunostimulatory RNA (isRNA) in CBA/LacSto mice with hepatocellular carcinoma G-29. We show that the isRNA induces interferon-α production in mice, inhibits tumor growth, and efficiently reduces the metastases area in the liver, kidneys, and heart of mice with HCC G-29.

## Methods

### RNA

Oligoribonucleotides (strand 1: 5′-AAAUCUGAAAGCCUGACACUUA-3′ and strand 2: 5′-GUGUCAGGCUUUCAGAUUUUUU-3′) were synthesized on an automatic ASM-800 DNA/RNA synthesizer (Biosset) using ribo-β-cyanoethyl phosphoramidites (Glen Research). After standard deprotection, oligoribonucleotides were purified using denaturing polyacrylamide gel electrophoresis (PAGE) and were then isolated as sodium salts. Oligoribonucleotides were characterized by MALDI-TOF mass spectra on REFLEX III (Bruker Daltonics, Germany). isRNAs were annealed at a concentration of 50 μM in a buffer containing 30 mM HEPES-KOH (pH 7.4), 100 mM sodium acetate, and 2 mM magnesium acetate.

High purity polyinosinic-polycytidylic acid (poly(I:C)) (less than 1% free nucleotides) was purchased from Sigma-Aldrich.

### Mice and injection of isRNAs

All animal procedures were carried out in accordance with the protocols approved by the Bio-ethics committee of the Siberian Branch of the Russian Academy of Sciences and recommendations for proper use and care of laboratory animals (European Communities Council Directive 86/609/CEE). We used male CBA/LacSto (CBA) mice from vivarium of the Institute of Cytology and Genetics SB RAS. Mice were 10–14 weeks old at the beginning of the experiments with an average weight of 23–27 g. Mice were housed in groups of 8–10 individuals in plastic cages, and had free access to food and water; daylight conditions were normal.

isRNA and poly(I:C) were complexed with Lipofectamine 2000 (Invitrogene) according to the manufacturer’s protocol. Briefly, 10 μg per mouse of isRNA or poly(I:C) was mixed with 35 μl of Lipofectamine in 200 μl of OptiMEM medium and incubated at room temperature for 20 min, and then the preparation was injected intraperitoneally or intravenously into mice.

### Analysis of IFN-α and IL-6 levels in mouse blood serum

10–14-week-old male CBA mice were injected intraperitoneally with 200 μl of isRNA/Lipofectamine complexes in sterile OptiMEM medium and the blood was collected 1, 6, 10 or 16 hours after the injection via head-clipping. The serum was prepared from the whole blood by coagulation for 30 min at 37°C and subsequent centrifugation, and the levels of IFN-α and IL-6 were measured using sandwich ELISA kits (BD Biosciences) in accordance with the manufacturer’s instructions. Samples were measured in triplicate.

### Tumor treatment

Transplantable Hepatocarcinoma-29 (G-29) tumor was used throughout these experiments. It is maintained in the vivarium of ICG SB RAS by regular passages in CBA mice. The solid form of the tumor was used in these experiments. 2 × 10^5^ tumor cells in a volume of 100 μl were transplanted into the right thigh muscles of 40 male CBA mice. After tumor transplantation, the animals were divided into experimental and control groups and then were kept in their compartments until the end of the experiment. For tumor treatment, on days 2, 5, 8, and 12 after tumor transplantation, mice received intraperitoneal injections of isRNA/Lipofectamine complexes in sterile OptiMEM. Control mice were injected with the same volumes of vehicle. Tumor volume was measured with calipers and calculated using the formula π/6 × (length × width × height). On the 24^th^ day after tumor inoculation, three mice from each group were taken out of the experiment by dislocation of cervical vertebrae. The organs of mice and the primary tumors were fixed in 4% formaldehyde solution in DMEM, and processed for paraffin sectioning by standard protocols. The remaining tumor-bearing mice were kept for the study of the survival rate.

### Microscopy

Paraffin cross-sections of the primary tumor were stained with hematoxylin/eosin and with the Mallory staining according to standard protocols. Images were obtained using a microscope DM2500 equipped with a digital camera DFC420 (Leica, Germany). The percentage of the metastases area was determined relative to the total area of sections using Adobe Photoshop Software from five random fields of view (magnification of lens 10×), and the confidence interval with parameter α = 0.05. Metastases in the heart had a strongly elongated shape that did not allow applying this method for the evaluation of their relative area. The area of metastases in the heart was determined using the following formula: X = (20 × l × n/10^6^) × 100, where the average width of the metastasis in mice hearts from all groups was obtained from direct measurements and set as 20 μm. l and n were the average length and number of metastases per mm^2^ of cross section of the mice heart in each group, respectively.

### Statistics

The statistical significance of the differences in IFN-α production was determined using the two-tailed Student’s t-test (data are expressed as means ± SD). The nonparametric Mann–Whitney U test was used for the analysis of the tumor size and metastases area to compare the mean values between two groups (data are expressed as means ± SEM). Differences were considered statistically significant for p < 0.05.

## Results

### Cytokine induction by isRNAs in mice

In the present study we used the most effective among previously tested [25] immunostimulatory isRNA molecules of 19-bp long with 3-nt overhangs at the 3′-ends. As the positive control, poly(I:C), which is known to induce an interferon response, was used [27].

**Figure 1:**
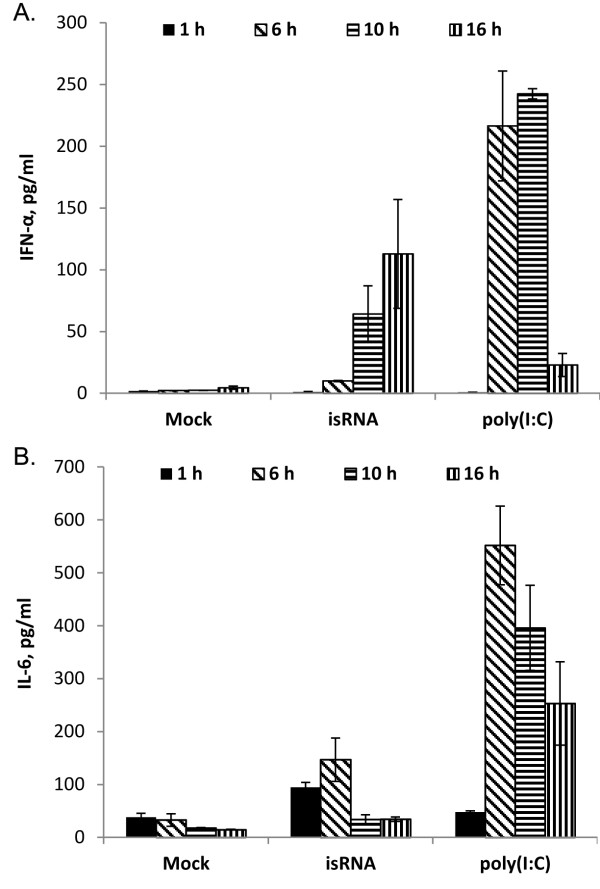
**Stimulation of the innate immune response by isRNA and poly(I:C) in vivo.** СBA/LacSto mice were intraperitoneally injected with isRNA (10 μg per mouse) or poly(I:C) (10 μg per mouse) in complex with Lipofectamine 2000. Serum IFN-a **(A)** and IL-6 **(B)** levels were measured by ELISA 6 h 1, 6, 10, and 16 hours after injection. Values for Lipofectamine-treated mice represent the Mock. The data represent means ± standard deviation (SD) calculated from measurements from at least three mice.

The levels of IFN-α and IL-6 in mouse blood serum after intraperitoneal administration of isRNA or poly(I:C) in CBA/LacSto mice were assayed to determine the immunostimulatory potential of isRNA. Groups of mice were injected with isRNA or poly(I:C) (10 μg per mouse) complexed with Lipofectamine, or with Lipofectamine only. The blood samples were collected 1, 6, 10, and 16 hours post-injection to measure the levels of IFN-α and IL-6. The results reveal (Figure 1) that both isRNA and poly(I:C), induce IFN-α and IL-6 secretion in mouse blood serum. isRNA induces 15-fold increase of IFN-α level in serum 10 hours after injection (Figure 1A), and the maximum level of IFN-α synthesis was observed 16 hours after injection, up to a 25-fold increase relative to the level in the mice injected with Lipofectamine (“Mock”). A different time-course was observed for poly(I:C). Thus, the maximum level of IFN-α was observed 6 – 10 hours post-injection and reached the level 2.2-fold higher than that for isRNA. The time-courses of the activation of IL-6 synthesis (Figure 1B) were also different for isRNA and poly(I:C). Thus, the isRNA induces 2.5 - 4.5-fold increase of IL-6 level relative to the level in Mock-treated mice 1 – 6 hours after injection, and reached the initial level by 10 h. The poly(I:C) induces more strong and prolonged increase of IL-6 level up to ~17-fold relative to the level in Mock-treated mice, 6 – 16 hours post-injection. Injections of Lipofectamine (“Mock”) without isRNA also induced a ~2-fold increase of IL-6 level 1–6 hours post-injection.

### Antitumor effect of isRNA

**Figure 2:**
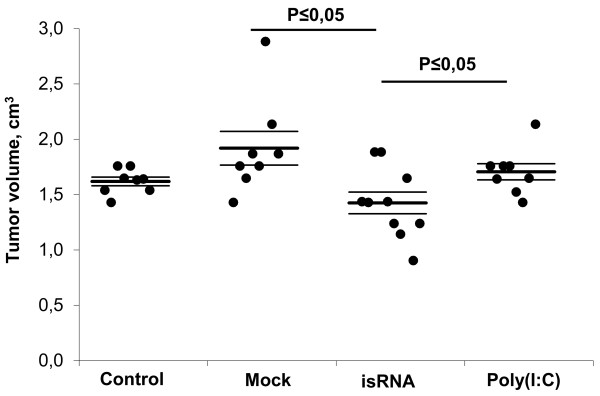
**Influence of isRNA injections on HCC primary tumor growth.** CBA/LacSto mice were intramuscularly injected with G-29 cells and treated with isRNA or poly(I:C) i.p. injections. The data represent means ± standard error of the mean (SEM) at day 22 (n = 8–10). *P* ≤ 0.05 according to Mann–Whitney test.

Antitumor and antimetastatic properties of isRNAs were investigated in the model of HCC G-29 tumor that is accompanied by the formation of multiple metastases in different organs of mice [28]. The experiments were performed according to the following scheme: G-29 cells (200,000 cells/mouse) were intramuscularly injected in the right thigh of mice. The intraperitoneal injections of isRNA or poly(I:C) complexed with Lipofectamine, as well as mock injections (Lipofectamine 2000), were performed at days 2, 5, 8, and 12 after tumor implantation. During the experiments, the size of the primary tumors was monitored each day. Data displayed in Figure 2 show that injections of isRNA caused reliable (Р < 0.05) inhibition of HCC G-29 tumor growth in comparison with the mock-treated group of mice. However, the difference in tumor size between isRNA-treated and untreated animals was not statistically relevant. It should be noted that in the group of mice treated with poly(I:C), the average tumor size was comparable with the tumor size in the untreated animals.

**Figure 3:**
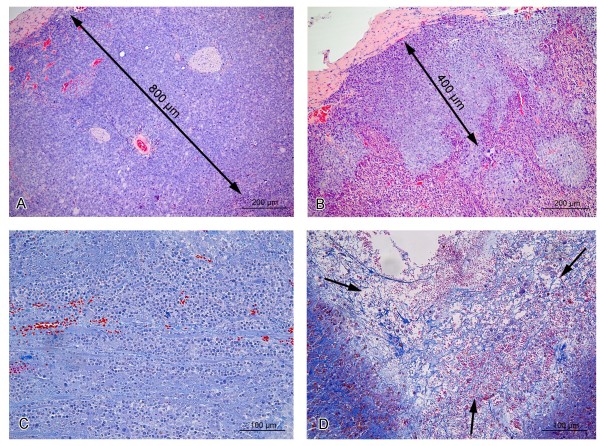
**Cross-sections of HCC G-29 primary tumors 22 days after tumor inoculation.** Peripheral zone of primary tumor in untreated **(A)** and isRNA-treated **(B)** mice: arrows show zone of undifferentiated cells. **C**, **D** - central area of primary tumor in untreated **(C)** and isRNA-treated **(D)** mice: necrosis and foci of cell death are indicated by arrows. Paraffin sections, Mallory’s trichrome staining.

Since the primary tumor is the source of HCC cells metastasizing to other organs, it was interesting to study the influence of isRNA treatment on the morphology of the primary tumors. Paraffin sections of primary tumors were prepared according to standard protocols and studied by light microscopy. The primary tumor under a connective tissue capsule was composed of two distinct zones formed by different cellular elements: peripheral (Figure 3A, B) and central (Figure 3C, D). The peripheral zone was located under the skin and contained elongated cells (8–10 μm) with a large nucleus occupying most of the cell, and a light cytoplasm. The boundaries of the cells were fuzzy. These cells actively divided, and a large number of mitoses were seen in the sections. The peripheral zone of the primary tumor was characterized by good vascularization and a small intercellular space (Figure 3A, B). Presumably, from this area, cells entered the bloodstream and subsequently developed metastases in the visceral organs. The central zone had lower vascularization and cells were arranged in bundles of 3–5 cells separated by thin layers of connective tissue. Mitoses were absent. The cells in this zone were larger (>10 μm), and round-shaped with a round nucleus surrounded by eosinophilic cytoplasm. Binuclear cells were often found, and sometimes there were multinucleated cells. The boundaries of the cells were clearly distinguishable, and widened intercellular spaces were seen along many apoptotic cells and necrotic foci (Figure 3C, D).

Morphometric analysis of primary tumor cross-sections demonstrated that isRNA treatment caused a more than twofold decrease of the width of the peripheral zone in comparison with those in untreated or mock-treated mice. Thus, the average width of the peripheral zone in isRNA-treated mice was 400 μm (Figure 3B) versus 800–900 μm for control mice (Figure 3A) and 700–4000 μm for mock-treated mice. In the peripheral zone of mice injected with isRNA, the number of mitoses was reduced in comparison with the mice from control groups from 15 to 8 per mm^2^ of cross sections. There were the large focal necroses that formed several large cavities in the central zone of primary tumors of isRNA-treated mice (Figure 3D), while in other groups the cavities were much smaller (Figure 3C). It should be noted that injections of poly(I:C) also caused a more than twofold decrease in the number of dividing cells (to 6.5 per mm^2^ of sections) in the peripheral zone of the primary tumor. However, the width of the peripheral zone of tumor in poly(I:C)-treated mice was highly varied (up to 1.5 mm).

**Figure 4:**
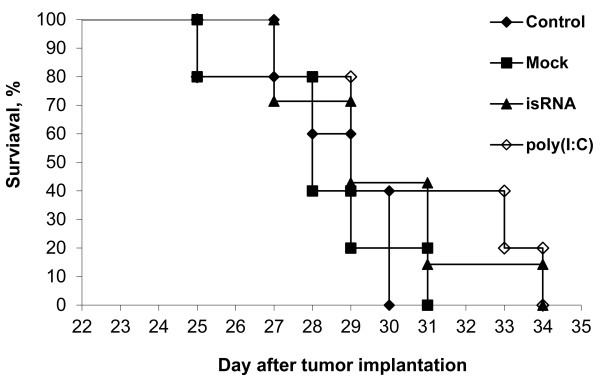
**The survival rate of mice after inoculation of HCC G-29 cells.** Each group consisted of 7 mice.

To assess the therapeutic efficacy of isRNA monotherapy, we compared the survival of the mice in different experimental groups. It was shown (Figure 4) that isRNA treatment increased the survival time by 5% in comparison with the mice from untreated and mock-treated groups. Thus, in the control and mock-treated groups of mice, the average life expectancy was 28.8 ± 0.6 and 28.2 ± 0.9, respectively. In the isRNA- or poly(I:C)-treated groups of mice, the average life expectancy was 29.7 ± 0.9 and 30 ± 1.6, respectively.

### Antimetastatic effect of isRNA

**Figure 5:**
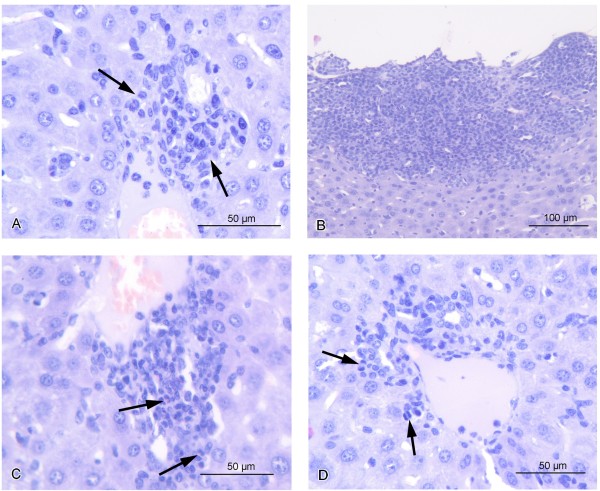
**Liver of CBA/LacSto mice 22 days after HCC G-29 cells inoculation showing internal and surface metastases. A**. Untreated mice. Arrows show the typical metastasis. In the blood vessel (*) painted plasma and a deformed erythrocytes can be seen. **B**. Large surface metastasis in liver of poly(I:C) treated mice. **C**. Eosinophils (arrows) and **D**. mitoses (arrows) in liver of mice after isRNA injections. Paraffin sections, hematoxylin and eosin staining.

**Figure 6:**
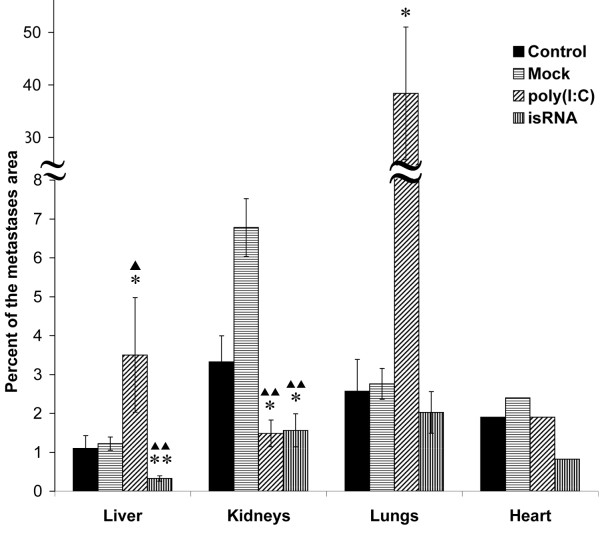
**Treatment of HCC G-29-bearing mice with isRNA reduces the metastases area in different organs of mice.** The ratio of the total metastases area to the total area of the organ was determined by microscopic analysis of organs sections, prepared at day 22. The data represent means ± SEM from five microscopic fields. Statistically significant differences between experimental groups and untreated group (control) are indicated by asterisks. **, P < 0.01; *, P < 0.05. Statistically significant differences between experimental groups and Lipofectamine-treated group (Mock) are indicated by triangles. ▲▲, P < 0.01; ▲, P < 0.05; Mann–Whitney U test. The area of metastases in the heart was evaluated using the average length of metastases and the mean number of metastases per mm^2^ as described in the Methods section.

Morphometric analysis to measure the average metastases area relative to the total area in different visceral organs was performed to evaluate antimetastatic effects of isRNA in mice with HCC G-29. Morphology of metastases in different organs of all mice visually did not differ (Figure 5), while the size and number of metastases per mm^2^ of section in isRNA-treated mice significantly decreased in comparison with control groups of mice and poly(I:C)-treated mice (Figure 6) indicating the retardation of metastases development. The presence of mitotic cells (Figure 5D), eosinophils (Figure 5C), and neutrophils in the sections of isRNA-treated mice indicates signs of the activation of the immune response. It should be noted that leukocytes were not observed in the organ sections of poly(I:C)-treated mice.

Morphometric analysis showed evident reduction in the number of metastases and their average size in organs of isRNA-treated mice in comparison with organs of the untreated, mock-treated, and poly(I:C)-treated animals (Figure 6). The injections of isRNA lead to a twofold decrease of the total area of metastases in the kidneys and the heart, and to a threefold decrease of metastases area in the liver (Figure 6). Notably, the injections of poly(I:C) in mice caused a 15-fold increase of metastases area in the lungs and a threefold increase in the liver in comparison with the untreated or mock-treated mice. In the kidneys of mock-treated mice, a twofold increase of metastases area in comparison with the untreated mice was observed.

## Discussion

The present study shows that 19-bp RNA duplex with 3-nt overhangs at the 3′-ends of specific sequence displays immunostimulatory activities in mice with hepatocellular carcinoma. This isRNA is one nucleotide longer than canonical siRNAs and has no substantial homology with mRNAs from mice and humans, and hence this isRNA cannot change the gene expression pattern via an RNA interference mechanism. Analysis of immunostimulatory activity of isRNA *in vivo* demonstrates that isRNA strongly stimulates the synthesis of IFN-α by up to 25-fold and only a 4.5-fold increase of IL-6 level relative to the level in Mock-treated mice (Figure 1A) after intraperitoneal injections. Although the control type 1 interferon inducer poly(I:C) [27] activates the synthesis of IFN-α more effectively than isRNA, it induces as high as an 17-fold increase of IL-6 level (Figure 1B). The data obtained are in agreement with our previous results [25] demonstrated that isRNA after intravenous administration mainly induces the synthesis of IFNα and to a lesser extent the synthesis of IL-6. It should be noted, that after i.p. and i.v. administration of isRNA or poly(I:C) no increase of tumor necrosis factor-α (TNF-α) level was observed in mouse blood serum (data not shown). The fact that isRNA induces the synthesis of type 1 interferon but not pro-inflammatory cytokines is important for the evaluation of the inducer as a potential adjuvant, since the inflammatory microenvironment contributes to the development of hepatic fibrosis, cirrhosis, carcinogenesis, and eventually tumor metastasis [29,30]. Pro-inflammatory cytokines TNF-α and IL-6 were found to be the main mediators of HCC invasion [31]. Similar results were reported by other groups [26,32-34]; these data show that 6–7 hours after injection into mice of different types of immunostimulatory siRNA with mixed functions complexed with cationic liposomes, a systemic immune response was induced, accompanied by IFN-α, IL (interleukin)-6, and/or IFN-γ production.

In the present study, we show that isRNA applied four times at the beginning of tumor development reliably (P < 0.05) inhibits primary tumor growth in mice as compared to the mock-treated group. Furthermore, injections of isRNA significantly increased the intensity of necrotic processes in the center of the primary tumor, and decreased by twofold the width of the undifferentiated peripheral zone and the number of mitotic cells in this zone. We used the following scheme of the treatment: four i.p. injections of isRNA every 3 days starting on the 2^nd^ day after tumor inoculation. Similar treatment regimens (three systemic injections with 3-day intervals) were used by two groups of authors [34,35]. Thus, our results reveal that isRNA retained the antitumor properties, affecting tumor growth, but the applied scheme of isRNA application is not sufficient for the restriction of the tumor growth. Prolonged treatment with isRNA and/or optimization of the scheme of monotherapy is required to achieve effective suppression of tumor growth. Probably, this preparation can display better results as a part of combined treatment with chemotherapy.

The antimetastatic effects of isRNA were more evident. Our results demonstrate that isRNA efficiently reduces the metastases area in the liver, kidneys, and heart of CBA/LacSto mice bearing HCC G-29. The incidence of mitosis, together with the small size of metastases, indicates that the metastases in isRNA-treated mice are at an earlier stage of development than that in the control group of mice. Infiltration of the tumor with eosinophils and neutrophils was observed only in isRNA-treated animals, which suggests an activated immune response. However, despite the fact that isRNA displays immunostimulatory, antitumor, and antimetastatic properties, the lifespan of isRNA-treated mice was not significantly increased because of the ineffective suppression of primary tumor growth.

It should be noted, that the injections of poly(I:C) complexed with Lipofectamine, or Lipofectamine alone resulted in an increase of metastases area in some organs and a slight increase in tumor volume in comparison with the untreated mice. Although poly(I:C)/Lipofectamine complex effectively induces IFN-α synthesis, it also leads to prolonged and efficient increase of pro-inflammatory cytokine IL-6 level. Several studies have shown that in case of HCC the development of inflammatory response contributes to the development of cirrhosis, carcinogenesis, and eventually tumor metastasis [29,30]. Lipofectamine is a lipid-based carrier and itself may also induce inflammation [36], that is far less toxic than the poly(I:C)/ Lipofectamine complexes, but they are not without overt toxicity.

It has been shown in several studies that activation of innate immunity by the addition of RIG-I agonist triphosphate to the 5′-ends of oncogene-specific siRNAs [34], or by conjugation of siRNA with CpG oligonucleotide, a potent TLR9 agonist [37,38], can potentiate siRNA antitumor effects. Antitumor activity is employing via the induction of type I IFNs, the activation of tumor-resident macrophages, and the restoration of normal immune function. In a recent study, it has been shown by Khairuddin et al. that activation of the innate immune response by siRNAs has significant antitumor effects against HPV-driven tumors, even in the absence of a specific gene target [35]. These findings imply the potential prophylactic and therapeutic use of immunostimulatory siRNAs as adjuvants.

In the present study, we applied 19-bp RNA duplex with 3-nt overhangs at the 3′-ends in complex with Lipofectamin for the therapy of HCC G-29 bearing mice. Based on published data, at least 4 signaling pathways may recognize small RNA molecules and induce production of type I IFNs and pro-inflammatory cytokines, including the RIG-I/MDA5 pathway, the TLR3 pathway, the TLR7/8 pathway, and the PKR pathway. RIG1 recognizes blunt-ended dsRNA molecules with a 5′-triphosphate that are over 20 bp long [39], and MDA5 is activated by long dsRNA [40]. Since our isRNA is 19 bp duplex with 3-nt overhangs and does not contain blunt ends or triphosphate residues, it was supposed that it activate innate immunity trough RIG1/MDA5-independent pathway. The isRNA under study may, on the one hand, activate PKR, leading to PKR auto phosphorylation, and on the other hand, enter the endosome by Lipofectamine and recognized by TLR3, 7/8. Both the PKR activation and TLR3, 7/8 signaling will stimulate production of type I IFNs and pro-inflammatory cytokines. Several studies have shown that small RNAs complexed with cationic delivery vehicles can stimulate the innate immune response by activating TLR3 [41,42], TLR7/8 [26,32], and PKR [43,44], or by involving some of these cellular sensors [45,46].

Thus, our results and results of different studies [27,34,36,38] indicate that activation of innate immunity by immunostimulatory nucleic acids and their analogs possess antitumor and antimetastatic properties in tumor-bearing mice. These properties were proved to depend on the type I IFN signaling pathway, since anti-metastatic properties of the drugs were significantly decreased in mice deficient for the IFN-α/β receptor [47].

## Conclusions

Our results show that 19-bp-long isRNAs with 3-nt overhangs at the 3′-ends induce IFN-α (Figure 1), but not proinflammatory cytokines IL-6 and TNF-α secretion in CBA/LacSto mice [25]. It is very important for HCC treatment to use type I IFN inducers, because in many cases HCC develops on a background of chronic inflammatory liver disease (hepatitis B and C, cirrhosis) and secretion of pro-inflammatory cytokines could additionally complicate the course of disease. IFN therapy, however, significantly suppresses development of HCCs on the background of chronic hepatitis and liver cirrhosis [14-16]. Our data revealed that isRNA displays efficient immunostimulatory and antimetastatic properties in mice with HCC. Moreover, isRNA treatment leads to a decrease in the pool of undifferentiated cells in HCC G-29 primary tumor nodes of mice. Thus, the studied isRNA can be considered as a potential adjuvant for the therapy of HCC and other immunosuppressive oncological diseases.

## Abbreviations

HCC: Hepatocellular carcinoma; siRNAs: Small interfering RNAs; isRNA: ImmunoStimulatory RNA; IFN: Interferon; RIG-I: Retinoic acid inducible gene-I; PKR: Interferon-induced, double-stranded RNA-activated protein kinase; IP-10: IFN-γ-inducible protein 10; PBMCs: Peripheral blood mononuclear cells.

## Competing interests

The authors declare that they have no competing interests.

## Authors’ contributions

Conceived and designed the experiments: TK, EC. Performed the experiments: TK, LK, NP, VN, VK. Analyzed the data: TK, EZ, ER, EC. Performed microscopic analysis: EZ, ER. Wrote the paper: TK, EC. Revised the manuscript: EC, MZ. VV participated in discussion and data interpretation. All authors read and approved the final manuscript.

## Pre-publication history

The pre-publication history for this paper can be accessed here:

http://www.biomedcentral.com/1471-2407/14/338/prepub
